# Barriers and facilitators of interventions for improving antiretroviral therapy adherence: a systematic review of global qualitative evidence

**DOI:** 10.7448/IAS.19.1.21166

**Published:** 2016-10-17

**Authors:** Qingyan Ma, Lai Sze Tso, Zachary C Rich, Brian J Hall, Rachel Beanland, Haochu Li, Mellanye Lackey, Fengyu Hu, Weiping Cai, Meg Doherty, Joseph D Tucker

**Affiliations:** 1 University of North Carolina Project-China, Guangzhou, China; 2 Guangzhou Eighth People's Hospital, Guangzhou, China; 3 Center for Medical Humanities, Zhongshan School of Medicine, Sun Yat-sen University, Guangzhou, China; 4 Institute for Global Health and Infectious Diseases at UNC-Chapel Hill, Chapel Hill, NC, USA; 5 Department of Health, Behavior and Society, Johns Hopkins Bloomberg School of Public Health, Johns Hopkins University, Baltimore, MD, USA; 6 Global and Community Mental Health Research Group, Department of Psychology, University of Macau, Macau, China; 7 Department of HIV/AIDS, World Health Organization, Geneva, Switzerland; 8 Department of Epidemiology, School of Public Health, Shandong University, Jinan, China; 9 Spencer S. Eccles Health Sciences Library, University of Utah, Salt Lake City, UT, USA

**Keywords:** ART adherence, intervention, qualitative research, systematic review, health policy

## Abstract

**Introduction:**

Qualitative research on antiretroviral therapy (ART) adherence interventions can provide a deeper understanding of intervention facilitators and barriers. This systematic review aims to synthesize qualitative evidence of interventions for improving ART adherence and to inform patient-centred policymaking.

**Methods:**

We searched 19 databases to identify studies presenting primary qualitative data on the experiences, attitudes and acceptability of interventions to improve ART adherence among PLHIV and treatment providers. We used thematic synthesis to synthesize qualitative evidence and the CERQual (Confidence in the Evidence from Reviews of Qualitative Research) approach to assess the confidence of review findings.

**Results:**

Of 2982 references identified, a total of 31 studies from 17 countries were included. Twelve studies were conducted in high-income countries, 13 in middle-income countries and six in low-income countries. Study populations focused on adults living with HIV (21 studies, *n*=1025), children living with HIV (two studies, *n*=46), adolescents living with HIV (four studies, *n*=70) and pregnant women living with HIV (one study, *n*=79). Twenty-three studies examined PLHIV perspectives and 13 studies examined healthcare provider perspectives. We identified six themes related to types of interventions, including task shifting, education, mobile phone text messaging, directly observed therapy, medical professional outreach and complex interventions. We also identified five cross-cutting themes, including strengthening social relationships, ensuring confidentiality, empowerment of PLHIV, compensation and integrating religious beliefs into interventions. Our qualitative evidence suggests that strengthening PLHIV social relationships, PLHIV empowerment and developing culturally appropriate interventions may facilitate adherence interventions. Our study indicates that potential barriers are inadequate training and compensation for lay health workers and inadvertent disclosure of serostatus by participating in the intervention.

**Conclusions:**

Our study evaluated adherence interventions based on qualitative data from PLHIV and health providers. The study underlines the importance of incorporating social and cultural factors into the design and implementation of interventions. Further qualitative research is needed to evaluate ART adherence interventions.

## Introduction

High levels of antiretroviral therapy (ART) adherence are necessary to achieve viral suppression, prevent drug resistance [1] and reduce opportunistic infections and morbidity [2]. UNAIDS proposed the 90-90-90 goals to end the AIDS epidemic by 2030 [3]. The third goal focuses on achieving viral suppression among all those who receive ART [3]. However, only 30% of PLHIV achieve viral suppression in the United States [4] and PLHIV in many other countries around the world have problems with viral suppression.

A key to attaining this goal is improving ART adherence. The WHO defines *treatment adherence* as “the extent to which a person's behaviour – taking medications, following a diet and/or executing lifestyle changes corresponds with agreed recommendations from a healthcare provider” [5]. Barriers to improving adherence include availability and cost of ART [2], poor healthcare infrastructure [6], low individual willingness to change lifestyles [2,6] and conflicts between medical practice and traditional cultural values [7]. Although a range of interventions have been undertaken to improve ART adherence, worldwide ART adherence rates vary widely [5].

Previous systematic reviews have focused on quantitative assessment of interventions for improving ART adherence [2,8–13]. While quantitative reviews are important to evaluate data on the effectiveness of interventions, qualitative reviews are helpful to summarize data on participant and stakeholder experiences of interventions. Qualitative research provides useful information on personal experiences [14] in addition to social and cultural factors influencing ART adherence [15]. Qualitative research has been increasingly integrated into interventions focused on improving ART adherence [16,17].

A systematic review of qualitative evidence of interventions for improving ART adherence can help to better understand barriers and facilitators to interventions [18,19]. It is imperative to understand potential participant harms and benefits to more effectively address health equity and human rights [20]. Therefore, the purpose of this review was to synthesize the global qualitative evidence of interventions for improving ART adherence among PLHIV and to inform patient-centred policymaking.

## Methods

### Search strategy

Our search strategy was implemented on 8 February 2015 to identify eligible studies using search terms in English without date restriction. We followed PRISMA guidance [21] and completed the ENTREQ checklist for qualitative systematic reviews [22] (Supplementary File 1 and 2). Our study was registered in PROSPERO (CRD42015017248). The following 19 databases were searched: CENTRAL (Cochrane Central Register of Controlled Trials), EMBASE, LILACS, PsycINFO, PubMed (MEDLINE), Web of Science/Web of Social Science, CINAHL, British Nursing Index and Archive, Social Science Citation Index, AMED (Allied and Complementary Medicine Database), DAI (Dissertation Abstracts International), EPPI-Centre (Evidence for Policy and Practice Information and Coordinating Centre), ESRC (Economic and Social Research Council), Global Health (EBSCO), Anthrosource and JSTOR. Conference proceedings including the Conferences on Retroviruses and Opportunistic Infections (CROI), International AIDS Conference (IAC) and alternating year International AIDS Society (IAS) clinical meetings were searched from their inception dates (1993, 1985 and 2001, respectively). We contacted the researchers and relevant organizations and checked the reference lists for all included studies. After identifying and deleting duplicates, citations and abstracts were imported into EndNote X7.

### Study selection

Two reviewers (QM and ZR) independently screened 2840 titles, 1066 abstracts and 137 full texts. Standardized inclusion criteria screened for studies were as follows: (1) intervention was clearly described; (2) qualitative findings were reported; (3) qualitative methodologies were used in data collection and analysis; and 4) the qualitative data presented experiences, attitudes and acceptance of interventions to improve ART adherence among PLHIV and treatment providers. Qualitative studies in mixed methods research were also included. A third reviewer (HL) resolved discrepancies at the level of full text between the two reviewers.

### Quality assessment

Two reviewers (QM and ZR) assessed the quality of included studies using an adaptation of the Critical Appraisal Skills Programme (CASP) quality-assessment tool [23]. No studies were excluded on the basis of quality assessment. Quality assessment included the following domains: qualitative, context, reflexivity, methodology, data collection, data analysis and sufficiency in evidence. For example, *reflexivity* refers to whether the researchers critically examined their relationship with participants when designing research questions and data collection [23]. The overall quality assessment of *high*, *moderate* or *low* was based on independent evaluation by two reviewers with discussion until consensus was reached in the case of discrepancies.

### Data extraction

Two reviewers (QM and ZR) extracted the data using a standardized set of categories including the following: (1) primary source data (quotes from stakeholders in improving ART adherence interventions); (2) secondary source data (interpretation from qualitative research studies); (3) characteristics of the studies such as location of the research, study dates, type of intervention, analytical methodology, themes, HIV-infected key populations and the population from which the data was collected. The first reviewer (QM) reviewed all manuscripts and assessed data extraction completeness.

### Data synthesis

We used a thematic synthesis approach that was developed *a priori* [24]. All data were entered into a spreadsheet. Comparisons across different studies were made using thematic analysis. We conducted initial open coding on each relevant text. First, we identified the six intervention-specific themes and analyzed their policy implications. Next, we identified themes that cut across different types of interventions. For each individual study, we assessed their quality, relevance, region and study location, income of the country and intervention type.

Each qualitative review finding was assessed using the CERQual (Confidence in the Evidence from Reviews of Qualitative Research) approach. CERQual is a method to assess and describe how much confidence to place in the findings from systematic reviews of qualitative evidence [25,26]. It has been used in other meta-synthesis of qualitative evidence [27]. The CERQual approach includes four elements: (1) methodological limitations of the individual studies, (2) relevance to the review question, (3) coherence and (4) adequacy of data [28]. The methodological limitations of the individual studies contributing to each review finding were assessed using the modified CASP tool [23]. Relevance was assessed by evaluating the applicability of the review findings to the context (perspective, population, setting) of the review question. Coherence was assessed by the degree of similarity across multiple studies or by whether convincing explanations accounted for the variation across studies [28]. Adequacy was assessed through the thickness of data, the number of studies, the stratification of countries or regions and the income level of the country in each individual study. If a review finding was supported by enough details from multiple primary studies, we claimed that the data for this finding was adequate. Based on an overall assessment of methodological limitations, relevance, adequacy and coherence, the confidence in the evidence for each review finding was assessed as high, moderate, low or very low [28].

## Results

### Study characteristics

**Figure 1:**
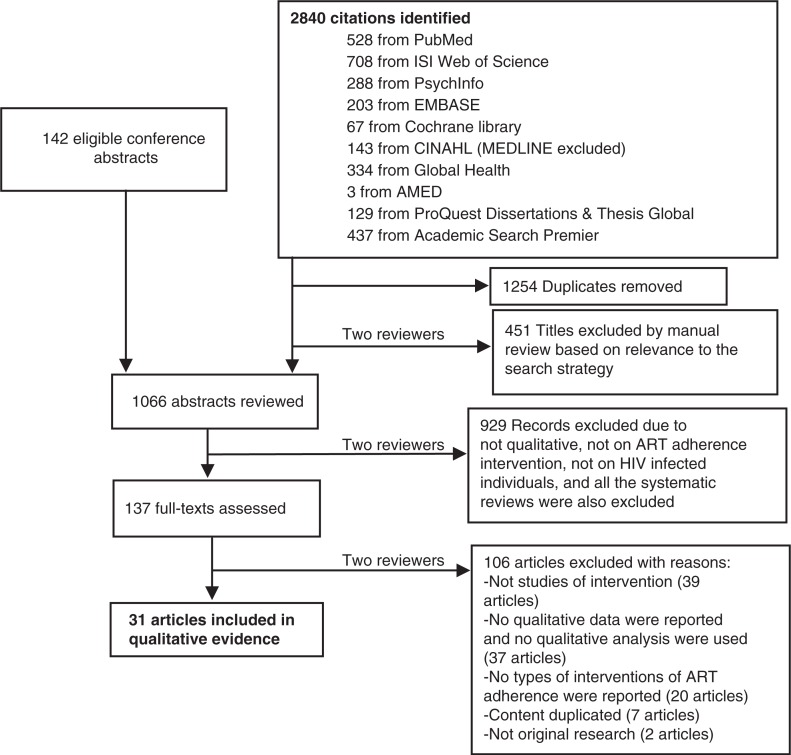
PRISMA flowchart of qualitative evaluations of antiretroviral therapy adherence interventions.

A total of 2982 titles and abstracts were identified for screening. Of these, 137 studies were examined at the level of full text (Figure 1). A total of 31 studies, including one dissertation [29] and 30 journal articles, were included for data extraction. Among the studies, 15 used mixed methods [16,17,29–41] and 16 were qualitative studies [15,42–56]. All studies were conducted in a single country. Twelve studies were conducted in high-income countries (HICs: Canada and United States) [33,34,40,44–46,48–52,56], 13 in middle-income countries (MICs: Brazil, China, India, Nigeria, Peru, Romania, South Africa, Swaziland, Thailand and Zambia) [15,17,30–32,36,38,41–43,47,53,54] and six in low-income countries (LICs: Mozambique, Rwanda, Tanzania, Uganda and Zimbabwe) [16,29,35,37,55]. The overall studies focused on children living with HIV (two studies) [30,54], adolescents (four studies) [43,45,48,53], adults (21 studies) [15,16–37,39–41,44,46,49–52,54,56] and pregnant women (one study) [55]. We identified 11 themes, including 6 themes related to types of intervention and 5 cross-cutting themes (Table 1). Our policy implication analysis revealed potential benefits, harms, equity and human rights, acceptability and feasibility for each intervention specific themes (Table 2). Table 3 summarized the quality, relevance, region and study location, income of the country and intervention type for the individual study.

**Table 1:** Summary of qualitative finding of interventions for ART adherence

Review finding	Relevant papers	CERQual confidence	Explanation of confidence in evidence assessment
*Task shifting* was acceptable by PLHIV and lay health workers to resolve the shortage of limited medical professionals, strengthen the relationship in the community, improve the psychosocial wellbeing of PLHIV and empower them to achieve better adherence. Proper training and compensation for lay health workers can better facilitate task shifting.	Alibhai 2014 Arem 2011 Born 2012 Dewing 2013 Nachega 2006 Rasschaern 2014 Root 2013 Shin 2011 Shroufi 2013 Thurman 2010 Torpey 2008	High	Eleven studies with minor to significant methodological limitations. Thick data from eight countries (three MICs, five LICs) and two regions (seven SSA, one LAC). High coherence and high relevance.
*Educating PLHIV and their families* was perceived to be acceptable and feasible by PLHIV. Some of them felt empowered to speak to health providers after participating in the intervention. In particular, PLHIV preferred educational interventions that conformed to local customs and were entertaining and simple.	Born 2012 Dima 2013 Fourney 2003 Holstad 2012 Lyon 2003 Magidson 2014 Watt 2011 Weiss 2006 Wong 2006	High	Nine studies with minor to significant methodological limitations. Thick data from five countries (one HIC, three MICs, one LIC) and three regions (three SSA, one Europe, five North America). High coherence and high relevance.
*Mobile phone text messages* were acceptable to PLHIV overall. They were well suited to reach marginalized populations. However, the duration of the mobile phone text messaging was relatively short and these studies did not evaluate post-intervention behaviours. Barriers noted included protecting privacy of mobile phone text messages and unintended disclosure of serostatus.	Costa 2012 Lin 2014 Montoya 2014 Moore 2013 Peterson 2014 Smillie 2014 Swendeman 2015 Weiss 2006	High	Eight studies with minor methodological limitations. Thick data from five countries (three MICs, two HICs) and three regions (two Asia, two North America, and one LAC). High coherence and high relevance.
*DOT* was acceptable to PLHIV. However, DOT providers needed to be familiar with PLHIV and develop a trusting relationship that ensures their privacy.	Garvie 2009 Lin 2014 Nachega 2006 Shin 2011	Moderate	Four studies with minor methodological limitations. Fairly thick data from three countries (United States, China, South Africa and Peru). Moderate coherence and high relevance.
*Medical professional outreach* was acceptable to PLHIV through providing counselling, psychosocial support and other social services for PLHIV, excluding the educational programmes mentioned above. However, medical professional outreach interventions could be enhanced through 1) maintaining the achievement of the intervention after it ends; 2) integrating the intervention into the overall welfare structure.	Anigilaje 2014 Nunn 2010 Rajabiun 2007 Rongkavilit 2010	Moderate	Four studies with minor methodological limitations. Fairly thick data from three countries (two MICs, one HIC) and three regions (two Asia, one North America and one SSA). Low relevance and moderate coherence.
*Complex intervention* using multiple interventions simultaneously was acceptable to PLHIV in one HIC. In contrast, another study conducted in an MIC suggested that administering and financing comprehensive intervention was challenging. Addressing administration and financing is critical for future adoption of the service model in order to maintain staff morale and commitment.	Lin 2014 Weiss 2006	Low	Two studies with minor methodological limitations. Limited and thin data from two countries (China and the United States). Moderate relevance and low coherence.
*Strengthening social relationships* among PLHIV and between non-infected community members was incorporated into peer education, task shifting and DOT. Strengthening social relationships can increase acceptability and feasibility of the intervention, improving psychosocial wellbeing of PLHIV and enhancing ARV adherence.	Alibhai 2014 Arem 2011 Born 2012 Dewing 2013 Garvie 2009 Lyon 2003 Nachega 2006 Rasschaert 2014 Root 2013 Shin 2011 Shroufi 2013 Thurman 2010 Torpey 2008 Weiss 2006	High	Fourteen studies with minor to significant methodological limitations. Thick data from nine countries (five LICs, three MICs, one HIC) and three regions (one North America, one LAC, seven SSA). High coherence and high relevance.
*Empowerment of PLHIV* refers to PLHIV's increased capacity, confidence and comfort to communicate with health providers and non-HIV-infected people about their serostatus and ART after participating in the intervention programmes. This cross-cutting theme emerged in seven intervention studies, including education, mobile phone text messaging and task shifting. Empowered PLHIV were more motivated to achieve better ART adherence.	Alibhai, 2014 Lyon 2003 Montoya 2014 Nachega 2006 Rasschaert 2014 Thurman 2010 Watt 2011	High	Seven studies with minor to significant methodological limitations. Thick data from seven countries (five LICs, two HICs) and two regions (two North America, five SSA). High coherence and high relevance.
*Compensation* was identified in studies of task shifting, education and complex interventions. Inadequate compensation for lay health workers and health providers were barriers for improving ART adherence.	Arem 2011 Born 2012 Lin 2014 Nachega 2006 Thurman 2010	High	Five studies with minor methodological limitations. Thick data from five countries (two MICs and three LICs) and two regions (one Asia and four SSA). High relevance and moderate coherence.
*Confidentiality* emerged as a key barrier in studies of DOT, task shifting and mobile phone text message interventions. PLHIV worried that participating in the intervention could lead to inadvertent disclosure of their HIV status. However, establishing a trusting relationship between PLHIV and DOT providers was helpful for overcoming concerns of intrusion into private life and reducing stigma.	Garvie 2009 Nachega 2006 Shin 2011 Swendeman 2015	High	Four studies with minor methodological limitations. Fairly thick data from four countries (United States, South Africa, Peru and India). High coherence and high relevance.
*Integrating religious beliefs into the intervention strategy*, for example Christianity and Buddhism, played an ancillary role in the interventions of mobile phone text messaging, task shifting and medical professional outreach. Integrating the religious belief into the intervention strategy can make the intervention more culturally appropriate and more acceptable to PLHIV.	Montoya 2014 Rongkavilit 2010 Root 2013	Moderate	Three studies with minor to moderate methodological limitations. Fairly thick data from three countries (United States, Thailand and Swaziland). High coherence and moderate relevance.

ART, antiretroviral therapy; DOT, directly observed therapy; LIC, low-income country; MIC, middle-income country; ARV, antiretroviral therapy; LAC, latin american countries; PLHIV, people living with HIV; SSA, sub-saharan africa; HIC, high income countries.

**Table 2:** Summary of evidence-to-policy implications for qualitative findings of ART adherence interventions

Intervention	Relevant papers	Potential harms	Potential benefits	Equity and human rights considerations	Feasibility	Acceptability
*(1) Task shifting*: shifting the taskfrom professional medical providers to community, family and lay people	Alibhai 2014 Arem 2011 Born 2012 Dewing 2013 Nachega 2006 Rasschaert 2014 Root 2013 Shin 2011 Shroufi 2013 Thurman 2010 Torpey 2008	Overall low potential harm. Inadvertent disclosure of HIV serostatus.	Helped resolve the shortage of medical professionals in resource-limited settings Strengthened the relationship between PLHIV and health providers Improved PLHIV's psychosocial wellbeing	Empowered PLHIV to achieve better adherence.	Feasible in resource-limited settings where human resources of medical professionals are limited	Overall accepted by PLHIV and CHW. Concerns about training, compensation and unintended disclosure of serostatus may limit the acceptability of the intervention.
*(2) Education*: improving knowledge of ART adherence through group information sessions and other media	Born 2012 Dima 2013 Fourney 2003 Holstad 2012 Lyon 2003 Magidson 2014 Watt 2011 Weiss 2006 Wong 2006	No potentials harms were identified.	Effectively improved knowledge Corrected misconceptions about ART adherence	Empowered PLHIV to talk to medical professionals and promoted equity and human rights.	Feasible for reaching people with low education, people who have mental health needs or adolescents	Generally accepted by PLHIV.
*(3) Mobile phone text message*: interventions using mobile phone text messages as reminders for ART adherence	Costa 2012 Lin 2014 Montoya 2014 Moore 2013 Peterson 2014 Smillie 2014 Swendeman 2015 Weiss 2006	Overall low potential harm. Privacy of mobile phone text messaging. Unintended disclosure of serostatus.	Low cost Convenient to read at any time Improved ART adherence	Technology can be a tool to educate and empower HIV infected individuals.	Well suited to reach marginalized populations, such as HIV-infected women drug users and incarcerated HIV-infected individuals	Overall accepted by PLHIV, except for unintentional disclosure of serostatus.
(4) DOT	Garvie 2009 Nachega 2006 Lin 2014 Shin 2011	Overall low potential harm. Intrusion into private life may increase chances of disclosing serostatus or increase stigma associated with HIV.	Improved ART adherence Improved psychosocial wellbeing	Intrusion into private life may be harmful for equity and human rights.	Feasible if DOT providers were familiar with PLHIV and had developed a trusting relationship to ensure the privacy of PLHIV	Accepted in circumstances where the PLHIV trusted and were familiar with the DOT providers.
*(5) Medical professional outreach*: interventions by medical professionals; outreach includes counselling, psychosocial support and other social services, excluding the educational programmes mentioned above	Anigilaje 2014 Nunn 2010 Rajabiun 2007 Rongkavilit 2010	Overall low potential harm. Discontinuation of the intervention due to the termination of the programme may set back the adherence.	Improved ART adherence among children, newly released prisoners, adolescents and other underserved population	No major implications for equity/human rights.	Feasible when the PLHIV were willing to participate in the intervention; the intervention can strengthen the bond between PLHIV, family members and society	Very well accepted in resource-limited settings.
*(6) Complex intervention*: multiple interventions implemented simultaneously, such as counselling, technology reminder, and social support groups	Lin 2014 Weiss 2006	Overall low potential harm. Lack of administrative and financial support will result in lower staff morale and commitment.	Improved ART adherence, PLHIV in high-income country can benefit from the intervention	No major implications for equity or human rights.	Feasible in high-income countries. Not quite feasible in middle- and low-income countries.	Very well accepted in high income countries.

ART, antiretroviral therapy; DOT, directly observed therapy; CHW, community health workers.

**Table 3:** Summary of the studies

First author	Quality	Relevance	Region	Location of research	Income	Type of intervention
Alibhai	High	High	Sub-Saharan Africa	Uganda	Low	Task shifting
Anigilaje	High	High	Sub-Saharan Africa	Nigeria	Middle	Medical professional outreach
Arem	High	High	Sub-Saharan Africa	Uganda	Low	Task shifting
Born	Moderate	High	Sub-Saharan Africa	Zambia	Middle	Task shifting
Costa	High	High	LAC	Brazil	Middle	Mobile phone text messaging
Dewing	Low	High	Sub-Saharan Africa	South Africa	Middle	Task shifting
Dima	High	High	Europe	Romania	Middle	Education
Fourney	High	High	North America	United States	High	Education
Garvie	High	High	North America	United States	High	DOT
Holstad	High	High	North America	United States	High	Education
Lin	High	Moderate	Asia	China	Middle	Complex intervention
Lyon	Moderate	High	North America	United States	High	Education
Magidson	High	High	North America	United States	High	Education
Montoya	High	Low	North America	United States	High	Mobile phone text messaging
Moore	High	High	North America	United States	High	Mobile phone text messaging
Nachega	High	Moderate	Sub-Saharan Africa	South Africa	Middle	Task shifting DOT
Nunn	High	Low	North America	United States	High	Medical professional outreach
Peterson	High	Moderate	North America	United States	High	Mobile phone text messaging
Rajabuin	High	Low	North America	United States	High	Medical professional outreach
Rasschaert	High	High	Sub-Saharan Africa	Mozambique	Low	Task shifting
Rongkavilit	High	Moderate	Asia	Thailand	Middle	Medical professional outreach
Root	Moderate	Moderate	Sub-Saharan Africa	Swaziland	Middle	Task shifting
Shin	High	High	LAC	Peru	Middle	Task shifting
Shroufi	High	Moderate	Sub-Saharan Africa	Zimbabwe	Low	Task shifting
Smilie	High	High	North America	Canada	High	Mobile phone text messaging
Swendeman	High	High	Asia	India	Middle	Mobile phone text messaging
Thurman	Moderate	Moderate	Sub-Saharan Africa	Rwanda	Low	Task shifting
Torpey	Moderate	High	Sub-Saharan Africa	Zambia	Middle	Task shifting
Watt	High	High	Sub-Saharan Africa	Tanzania	Low	Education
Weiss	High	High	North America	United States	High	Complex intervention
Wong	Low	High	Sub-Saharan Africa	South Africa	Middle	Education

DOT, directly observed therapy; LAC, latin american countries.

### Qualitative synthesis

Twenty-three studies provided detailed experiences of PLHIV [15,17,29,32–37,39,40,44–46,48–56]. Thirteen studies explored the experiences of healthcare providers and lay health workers in addition to the experiences of PLHIV [15,16,31,33,35,37,38,41–43,47,54,55]. Table 1 presents the summary of qualitative findings and CERQual confidence assessments.

### Types of intervention

#### Task shifting (11 studies, high CERQual confidence)

Adherence interventions focused on task shifting were only identified in LIC and MIC settings [15,29,16,31,35–38,42,54,55]. The tasks shifted included adherence support and counselling [15,16,29,35,36,38,42,55], education [31], directly observed therapy (DOT) [54] and case management [37]. These tasks were shifted from professional medical providers to community, HIV-infected peers and laypeople. Five studies evaluated task shifting to the community level [29,35–37,54]. Five other studies evaluated task shifting to HIV-infected peers [15,16,31,38,55] and one other study evaluated task shifting to laypeople [42]. Task-shifting studies focused on pregnant women living with HIV [55], impoverished adults and children living with HIV [54] and PLHIV in general [15,16,29,31,35–38,42]. These studies indicated that task shifting reduced the shortage of medical professionals in LICs and MICs [16,31,38] and helped strengthen the relationship between PLHIV and health providers by building trust [15,35,54]. In addition, task shifting improved the psychosocial wellbeing of PLHIV [36,37,54,55]. Several studies reported that proper training for lay health workers should be a necessary part of task shifting [31,35–38]. One study from Mozambique further clarified that essential knowledge and problem-solving skills were more useful than disease-specific treatment literacy in training for lay health workers [35]. Our findings also indicated that task shifting can be better facilitated and accepted by integration into the overall health system [15,35,37].

#### Educating PLHIV and their families (Nine studies, high CERQual confidence)

The importance of educating PLHIV and their families on the knowledge of ART adherence was a key issue for improving adherence. Nine studies focusing on education were identified in HICs (five countries) [33,40,44,46,48], MICs (three countries) [31,41,43] and LICs (one country) [39]. These interventions improved knowledge of the ART adherence of adults living with HIV (*n*=359), people with low education [44], those with mental health needs [33] and adolescents (*n*=43) [43,48]. The interventions were implemented through peer education [31,48], group information sessions [39,40] and videos, music or comic books [41,44,46]. Educational programmes were well received by PLHIV. They felt their knowledge of ART treatment was improved. One participant living with HIV from the United States asserted that the educational intervention was useful because “I now feel responsible and like I should take more care of myself” [48]. Educational interventions also corrected misconceptions about ART adherence for PLHIV – for example, that they can still take the pill later if they forget [39]. PLHIV generally affirmed that interventions were acceptable and feasible. Some individuals often felt empowered to seek advice from health providers after obtaining more knowledge through participating in the intervention [39,46]. In particular, individuals preferred educational interventions that were entertaining [44,46], simple [44] and used familiar metaphors that were consistent with local cultural norms [41].

#### Mobile phone text messages (Eight studies, high CERQual confidence)

Interventions using mobile phone text messages for medication reminders were identified in eight studies in HICs (five studies) [34,40,49,51,56] and LIMCs (three studies) [17,32,47]. Overall, text message interventions were acceptable [17,34,51,56] and feasible [17,34,49,56] for PLHIV and low cost for health providers [32,34]. They were well suited for reaching marginalized populations, such as women (*n*=84) [32,56], people who use drugs (*n*=49) [34,47,49] and incarcerated individuals (*n*=24) [51]. Intervention participants reported that the text messages were an incentive to take care of themselves [32] and a reminder to take medication [17,32,49]. However, the duration of the mobile phone text-messaging intervention was relatively short (weeks to months) and participants preferred long-term interventions [32]. An additional shortcoming is that these studies did not evaluate post-intervention behaviours. Concerns about privacy of mobile phone text messages and unintended disclosure of serostatus were noted [17]. One participant from India stated: “No one in my family knows anything about my HIV status. So it would raise certain issues of embarrassment for me” [17].

#### Directly observed therapy (Four studies, moderate CERQual confidence)

DOT was identified in four studies of adherence intervention across high income countries (HICs) (one study) [45] and low and middle income countries (LMICs) (three studies) [15,47,54]. DOT was considered an acceptable intervention by adolescents (*n*=17) [49], impoverished adults (*n*=95) and children (*n*=13) [54], as well as by health providers for people who use drugs [47]. An impoverished adult living with HIV in Peru said: “most of all, for those of us who have had the support of … [the DOT team], they have made us more conscientious” [54]. Two studies suggested that having DOT providers familiarized with PLHIV could help cultivate trusting relationships, which were able to ensure the privacy of these individuals [45,54]. Another study in South Africa also illustrated that having a family or community member from a trusted source as a DOT provider was acceptable and an important part of the treatment support network [15].

#### Medical professional outreach (Four studies, moderate CERQual confidence)

Medical professional outreach was identified in four studies across HICs (two studies) [50,52] and LMICs (two studies) [30,53]. The outreach interventions included counselling [52,53], psychosocial support [30] and other social services [50], excluding the educational programmes mentioned above. In these interventions, medical professionals provided the outreach intervention to children living with HIV (*n*=33) and their caregivers [30], newly released prisoners (*n*=20) [50], adolescents (*n*=10) [53] and other underserved populations (drug users, homeless people and incarcerated individuals) (*n*=76) [52]. The outreach intervention helped participants integrate ART adherence into their routine lives [52], provided better family support [30], community support [50] and knowledge of ART adherence [53].

However, several barriers existed for the outreach interventions. The first one was lack of programme sustainability [30]. This barrier posed a potential harm for the intervention, as the termination of the intervention would set back ART adherence. The second barrier was the intervention not being well integrated into the existing welfare and social support system [50]. Outreach interventions work better if integrated into the overall welfare structure, such as providing stable housing to newly released prison inmates to reduce non-medical barriers to adherence [50].

#### Complex intervention (Two studies, low CERQual confidence)

A complex intervention is a study design combining multiple single interventions for simultaneous implementation. Two studies evaluated complex interventions focusing on counselling, mobile phone text messages and social support groups [40,47]. Each type of intervention had been evaluated in a previous section. These studies reported sharp differences between the acceptability and feasibility of complex interventions in HICs (United States) [40] and LMICs (China) [47]. The study from the United States reported that PLHIV benefited from the complex intervention, while the study from China reported that barriers to administering and financing the complex intervention may negatively impact its acceptability and feasibility for health providers.

### Cross-cutting themes

#### Strengthening social relationships (14 studies, high CERQual confidence)

Strengthening social relationships is a cross-cutting theme identified in HICs (three studies) [40,45,48] and LMICs (11 studies) [15,16,29,31,35–38,42,54,55]. This includes strengthening social relationships among PLHIV [16,31,35,38,55] and between PLHIV and their family or community [15,29,36,37,40,42,45,48,54]. Interventions, including education (three studies) [31,40,48], task shifting (10 studies) [15,29,16,35–38,42,54,55] and DOT (two studies) [45,54], utilized strategies strengthening social relationships to improve ART adherence. The review finding suggests that interventions strengthening social relationships increased the intervention acceptability and feasibility by PLHIV [33,35,45] and lay health workers [54,55]. Strengthened social relationships also improved the psychosocial wellbeing of PLHIV, as identified in a study of task shifting in Mozambique [35], a study of DOT in Peru [54] and a study of educational intervention in the United States [48]. This is the potential benefit of these interventions.

#### Empowerment of PLHIV (Seven studies, high CERQual confidence)

Empowerment of PLHIV refers to providing PLHIV with increased capacity, confidence and comfort in communicating with health providers and non-HIV infected people about their serostatus and ART after participating in the intervention programmes. This cross-cutting theme emerged in studies in HICs (two studies) [48,49], MICs (one study) [15] and LICs (four studies) [28,35,37,39]. This theme also encompasses interventions from task shifting (four studies) [15,29,35,37], to education (two studies) [39,48] and mobile phone text messages (one study) [49]. In one educational intervention among adolescents living with HIV in the United States, one adolescent participant said: “It was very comforting. [I felt] open to speak on subjects that were normally hard to talk about with non-HIV people” [48]. Empowerment of PLHIV through interventions has an important potential benefit. The empowered individual living with HIV is more motivated to sustain better adherence [15] and to seek medical advice from health providers and support from community members [35,39].

#### Compensation (Five studies, high CERQual confidence)

Compensation for lay health workers and health providers was identified as a cross-cutting theme only in studies conducted in LMIC settings [15,16,31,37,47]. The need for adequate compensation to motivate lay healthcare workers was identified in studies of task-shifting interventions (two studies) [15,37], educational interventions (one study) [31] and complex interventions (one study) [47]. Inadequate compensation for peer educators [31], limited financial support for family members of PLHIV [15] and community case managers [37] in task shifting, and improper financial compensation for health providers in complex intervention [47] could be barriers for implementing the interventions. However, one study of a task-shifting intervention in Uganda showed that no peer health workers quitting the study may be the indicator that the compensation was sufficient [16].

#### Confidentiality (Four studies, high CERQual confidence)

Concerns about loss of confidentiality associated with participating in adherence interventions emerged as a barrier in intervention implementation [15,17,45,54]. It is important to note that this barrier was reported in high income countries (HICs) [45], MIC [15], and LMIC [19,56] settings across the three well-established types of interventions, such as task shifting [15], DOT [45,54] and a mobile phone text message intervention [17]. Adolescents living with HIV and adults reported that participating in interventions could inadvertently reveal their HIV status [15,17,45,54]. The studies also suggested that establishing a trusting relationship between HIV and DOT providers was helpful for overcoming concerns of intrusion to private life and reducing stigma [45,54]. A community health worker who participated in a community-based DOT intervention in Peru said: “little by little, they trusted me, they confided in me, they spoke with me about so many experiences” [54].

Integrating religious beliefs into interventions (Three studies, moderate CERQual confidence).

The importance of integrating religious beliefs into the intervention designs was identified in three studies [36,49,53] in HIC [49] and LMIC [36,53] settings. In a mobile phone text message intervention for current or former drug users in the United States [49], Christian beliefs were incorporated into the adherence intervention text message, such as “God grant me the serenity to do this.” The intervention was acceptable to PLHIV. In a task-shifting intervention for PLHIV in Swaziland, religious aspects were integrated into community-based care. One participant of this intervention said: “[and they counsel that] whenever I take ART, I must also pray to God because He is the one who cares [about] our lives” [36].

## Discussion

We systematically reviewed the qualitative evidence on barriers and facilitators of interventions for improving ART adherence. We also assessed the potential harms and benefits of the interventions and their equity and human rights implications. Our qualitative review findings bring together the powerful voices of those living with and affected by HIV. In addition, using the CERQual approach is a methodological advance [25] that provides transparency in examining the confidence of review findings. Our results may help inform evidence-based intervention design and patient-centred public health policy.

Our review identified several types of adherence interventions that introduced concerns about confidentiality. The possibility of inadvertent disclosure of serostatus through participation in the intervention was reported in DOT interventions [45,54], task shifting interventions [15] and a mobile phone text message intervention [17]. None of these studies mentioned whether serostatus disclosure was captured as an adverse outcome, and two related quantitative evaluations of the same interventions also did not measure this adverse outcome [17,57]. Another quantitative evaluation mentioned that loss of confidentiality might be a minor barrier to participation in mobile phone-based interventions [58–60]. Future studies could improve implementation effectiveness by addressing this concern and by incorporating measures to ensure the confidentiality of participants [15,17].

Empowerment of PLHIV is a major benefit we identified in several types of adherence interventions, including task shifting, education and mobile phone text message interventions. PLHIV empowerment could have significant implications for health equity and was not previously evaluated by quantitative adherence reviews [2,10,11], although other reviews noted the potential for empowerment as a result of interventions [13,61,62]. One review of both quantitative and qualitative evidence of interventions for HIV-infected pregnant and postpartum women in sub-Saharan Africa highlighted the importance of empowerment for women living with HIV [61]. Our findings are consistent with these reviews: that participating in these interventions empowered PLHIV, gave them greater motivation to sustain better adherence [15] and provided them with more courage to seek medical advice from health providers and support from community members [35,39]. This finding is consistent with other interventions that formally incorporated empowerment of PLHIV into their intervention design and secondary outcomes [63–65]. This suggests that empowerment of PLHIV may be useful when designing and implementing ART adherence interventions.

Our review identified inadequate compensation for peer educators [31] and lay health workers [15,37] as a key barrier for ART adherence interventions in LMICs. Delayed payment or limited financial support for lay health workers may result in reduced morale, commitment and capacity for supporting task shifting. Another systematic review suggested that task shifting has substantial cost savings for LMICs [12], but those studies did not evaluate levels of adequate compensation for lay health workers in calculations for the total cost of successful interventions. This finding is consistent with the World Health Organization's suggestion that policymakers should consider how compensation structures can better account for the opportunity costs for health workers to better implement task-shifting needs [66]. Other studies also identified inadequate compensation as a potential barrier of task shifting in LMICs [67–69]. For ART adherence interventions, it is particularly important to integrate fair compensation into the initial intervention design to ensure health equity and the long-term commitment of community health workers.

Among intervention-specific themes, task shifting is an important intervention in LMICs. There are implications at policy, community and individual levels of the findings that task-shifting interventions were only conducted in LMICs. Building upon previous quantitative systematic reviews that examined the efficacy of task shifting [12,69], our qualitative evidence suggests that at policy level the implication is that the WHO's HIV ART guidelines were well received in LMICs [5], and this finding is consistent with another literature review of task shifting in resource-limited settings [62]. At the local community level, our qualitative evidence reported that task shifting reduced the shortage of medical professionals in LMICs, and community health workers and PLHIV had positive perceptions toward task shifting. At the individual level, the implication is that task shifting helped strengthen social relationships between PLHIV and their local communities and empowered PLHIV.

Our qualitative evidence also highlighted the importance of strengthened social relationships due to task shifting. The stronger social relationships provided more social support for adherence [15,36,55], contributed to better psychological wellbeing [29,16,33,54,70] and reduced HIV- and ART-related stigma [29,54]. Although perceived by PLHIV as favourable, during implementation of task shifting, inadequate training for lay health workers [15,31,37,38] emerged as a potential barrier. In addition, the long-term impact of task shifting was not addressed [12]. Given these findings, longitudinal research on task shifting may be useful [12,69].

There are several limitations to our study. First, all studies included used data from single interviews without follow-up observations. People's perceptions towards an intervention may change over the course of the intervention. Second, qualitative data were limited to pregnant women, children and adolescents and were not available among key populations such as sex workers and men who have sex with men (MSM). There was only one intervention targeting pregnant women [55], two interventions targeting children living with HIV [30,54] and four targeting adolescents [43,45,48,53]. Third, none of the adherence interventions focused on individuals with high CD4 counts. These individuals are increasingly important in the context of universal test and treat programmes. Fourth, only 9 out of 31 intervention studies specified poor adherence and difficulty in adhering to ART [34,40,42,45,48,49] and new ART initiators [38,39,56] in their recruitment criteria for intervention studies. Fifth, this review was not completely integrated with a quantitative review. Further systematic reviews of interventions would benefit from paired qualitative and quantitative evaluations that create comparable categories of intervention. Finally, we only included published studies and conference abstracts, excluding potentially useful grey reports and related materials.

## Conclusions

Our qualitative evidence suggests that strengthening PLHIV social relationships, empowering PLHIV and developing culturally appropriate interventions may facilitate adherence interventions. Our study indicates that inadequate training and compensation for lay health workers and inadvertent disclosure of serostatus by participating in the intervention are potential barriers for ART adherence interventions. These findings have several research and policy implications. From a research perspective, qualitative research brings in the voices of PLHIV and health providers, which extends the quantitative research by assessing equity and human rights implications. Future qualitative evaluations are needed to provide more evidence of interventions targeting pregnant women, children, adolescents, sex workers and MSM. These key populations are of important implication in promoting ART adherence and HIV treatment in general. Our data suggest the need for interventions targeted to those subgroups at greatest need, such as those with treatment failure and demonstrated poor adherence. Additional qualitative research can help inform the scale-up of effective interventions and guide the transition from intervention to sustainable and routine programmes. From a policy perspective, four implications stand out. First, our findings underline the importance of taking social and cultural factors into consideration when implementing ART adherence interventions. Second, training to ensure privacy and confidentiality in the context of an ART adherence intervention is essential for these types of programmes. Next, proper training and compensation for lay health workers and peer educators should be included during implementation. Finally, policymakers should consider how to maintain intervention effects over time. As universal test and treat strategies are increasingly implemented around the world, ensuring high levels of adherence will be critical to achieve the third and final UNAIDS 90-90-90 goal of achieving viral suppression [3].

## Supplementary Material

Barriers and facilitators of interventions for improving antiretroviral therapy adherence: a systematic review of global qualitative evidence
