# Supplementary material for: Barriers and facilitators of interventions for improving antiretroviral therapy adherence: a systematic review of global qualitative evidence
Source: J Int AIDS Soc. 2016 Oct 17;19(1):21166. doi: 10.7448/IAS.19.1.21166 (PMC5069281; doi:10.7448/IAS.19.1.21166)
Supplement: Barriers and facilitators of interventions for improving antiretroviral therapy adherence: a systematic review of global qualitative evidence [file JIAS-19-21166-s001.pdf]

Supplement 1. Search terms used in querying qualitative ARV adherence results

(Adherence[tw] OR adhere[tiab] OR nonadherence[tiab] OR “non adherence”[tiab] OR compliance[tw] ORcomply[tiab] OR noncompliance[tiab] OR “non compliance”[tiab] OR Retain[tiab] OR retention[tw] ORretained[tiab] OR Uptake[tiab])
AND
(ARV[tiab] OR ARVs[tiab] OR ART[tiab] OR HAART[tiab] OR antiretroviral[tiab] OR antiretrovirals[tiab] OR “anti-retroviral"[tw] OR “anti-retrovirals”[tiab] OR stavudine[tw] OR D4T[tiab] OR zidovudine[tw] OR AZT[tiab] OR azidothymidine[tw])
AND
((HIV[tw] OR “human immunodeficiency virus”[tiab] OR AIDS[tw] OR “acquired immunodeficiency syndrome”[tw] OR serostatus[tw] OR PLHIV[tiab] OR people living with HIV[tiab] OR persons living with HIV[tiab] OR PLWHA[tiab] OR persons living with HIV/AIDS[tiab] OR people living with HIV/AIDS[tiab] OR people living with AIDS[tiab] OR persons living with AIDS[tiab]) OR ((viral OR virally OR HIV) AND (suppress OR suppression OR suppressed)))
AND(((“semi-structured”[TIAB] OR semistructured[TIAB] OR unstructured[TIAB] OR informal[TIAB] OR “in-depth”[TIAB] OR indepth[TIAB] OR “face-to-face”[TIAB] OR structured[TIAB] OR guide[TIAB] OR guides[TIAB]) AND
(interview*[TIAB] OR discussion*[TIAB] OR questionnaire*[TIAB]))
OR (“focus group”[TIAB] OR “focus groups”[TW] OR qualitative [TIAB] OR ethnograph*[TIAB] OR fieldwork[TIAB] OR “field work”[TIAB] OR “key informant”[TIAB] OR participant observation[tiab] OR participant observations[tiab] OR anthropology[tw] OR anthropological[tiab] OR narrative[tiab] OR voice[tiab] OR story telling[tiab] OR storytelling[tiab] OR stories[tiab] OR grounded theory[tw]))
OR “interviews as topic”[Mesh] OR narration [Mesh] OR qualitative research [Mesh] OR "personal narratives as topic"[Mesh] OR “anecdotes as topic”[Mesh]

Supplement 2

Table 1. CASP adapted measurement for assessing quality of qualitative evidence

Question*		Yes	No
1	Is the study qualitative research?		
2	Is the study context clearly described?		
3	Is there evidence of researcher reflexivity?		
4	Is the sampling method clearly described and appropriate for the research question?		
5	Is the method of data collection clearly described and appropriate		

	to the research question?		
6	Is the method of analysis clearly described and appropriate to the research question?		
7	Are the claims made supported by sufficient evidence? i.e., did the data provide sufficient depth, detail, and richness?		

*Note: Questions adapted from CASP tool, from Critical Appraisal Skills Programme, (2006) Qualitative Appraisal Checklist for Qualitative Research.
